# Environmental determinants of access to shared sanitation in informal settlements: a cross-sectional study in Abidjan and Nairobi

**DOI:** 10.1186/s40249-023-01078-z

**Published:** 2023-04-10

**Authors:** Vitor Pessoa Colombo, Jérôme Chenal, Fred Orina, Hellen Meme, Jeanne d’Arc Amoin Koffi, Brama Koné, Jürg Utzinger

**Affiliations:** 1 École Polytechnique Fédérale de Lausanne, Lausanne, Switzerland; 2 Université Mohammed VI Polytechnique, Ben Guerir, Morocco; 3 Kenya Medical Research Institute, Nairobi, Kenya; 4 Centre Suisse de Recherches Scientifiques en Côte d’Ivoire, Abidjan, Côte d’Ivoire; 5 Université Péléforo Gon Coulibaly, Korhogo, Côte d’Ivoire; 6 Swiss Tropical and Public Health Institute, Allschwil, Switzerland; 7 University of Basel, Basel, Switzerland

**Keywords:** Diarrhea, Informal settlements, Safety, Sanitation, Urban morphology, Abidjan, Africa, Côte d’Ivoire, Kenya, Nairobi

## Abstract

**Background:**

Universal access to basic sanitation remains a global challenge, particularly in low- and middle-income countries. Efforts are underway to improve access to sanitation in informal settlements, often through shared facilities. However, access to these facilities and their potential health gains—notably, the prevention of diarrheal diseases—may be hampered by contextual aspects related to the physical environment. This study explored associations between the built environment and perceived safety to access toilets, and associations between the latter and diarrheal infections.

**Methods:**

A cross-sectional study was carried out between July 2021 and February 2022, including 1714 households in two informal settlements in Abidjan (Côte d’Ivoire) and two in Nairobi (Kenya). We employed adjusted odds ratios (a*OR*s) obtained from multiple logistic regressions (MLRs) to test whether the location of the most frequently used toilet was associated with a perceived lack of safety to use the facility at any time, and whether this perceived insecurity was associated with a higher risk of diarrhea. The MLRs included several exposure and control variables, being stratified by city and age groups. We employed bivariate logistic regressions to test whether the perceived insecurity was associated with settlement morphology indicators derived from the built environment.

**Results:**

Using a toilet outside the premises was associated with a perceived insecurity both in Abidjan [a*OR* = 3.14, 95% confidence interval (*CI*): 1.13–8.70] and in Nairobi (a*OR* = 57.97, 95% *CI*: 35.93–93.53). Perceived insecurity to access toilets was associated with diarrheal infections in the general population (a*OR* = 1.90, 95% *CI*: 1.29–2.79 in Abidjan, a*OR* = 1.69, 95% *CI*: 1.22–2.34 in Nairobi), but not in children below the age of 5 years. Several settlement morphology features were associated with perceived insecurity, namely, buildings’ compactness, the proportion of occupied land, and angular deviation between neighboring structures.

**Conclusions:**

Toilet location was a critical determinant of perceived security, and hence, must be adequately addressed when building new facilities. The sole availability of facilities may be insufficient to prevent diarrheal infections. People must also be safe to use them. Further attention should be directed toward how the built environment affects safety.

**Graphical Abstract:**

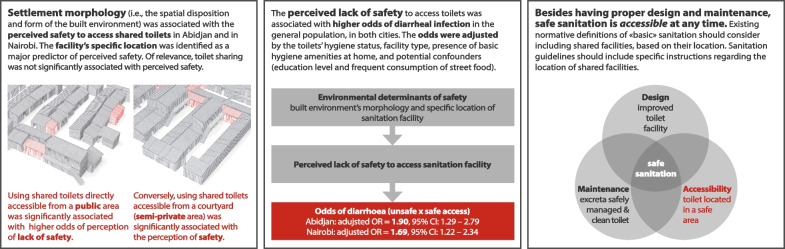

## Background

Sub-Saharan Africa is one of the fastest-urbanizing regions of the world [1]. This rapid urban growth brings about considerable challenges, such as the provision of services and infrastructures that are essential to public health. In 2020, more than 230 million people (50.2% of the urban population in this region) lived in “slum” households [2], i.e., households that lack one or more of the following: access to adequate sanitation facilities, eventually shared by a “reasonable” number of households; easy and affordable access to clean water; durable housing structure; sufficient living space; and security of tenure [3]. Considering previous debates on the term “slum” [4], which can be perceived as demeaning, we will henceforth refer to such areas as “informal settlements.”

Urban poverty and the ubiquity of improvised, informal settlements constitute a major challenge for global health and the pursuit of several of the United Nations’ Sustainable Development Goals (SDGs), particularly SDG 1 (no poverty), SDG 3 (good health and well-being), SDG 6 (clean water and sanitation), and SDG 11 (sustainable cities and communities). Beyond poverty, people living in informal settlements are exposed to specific “neighborhood effects” that exacerbate the risk of infectious and non-communicable diseases [5]. In this regard, the recent COVID-19 pandemic highlighted urban health inequities, recalling the need to improve access to essential services in informal settlements—notably, safe sanitation amenities—while addressing their specific social and economic vulnerabilities [6, 7]. In 2020, one out of five people in sub-Saharan African cities lacked access to improved sanitation [8], even though the latter is essential to prevent threatening diarrheal infections that represent the main contributor to the disease burden from water, sanitation, and hygiene (WASH) [9–12].

To monitor progress toward SDG 6, the World Health Organization (WHO)/United Nations Children’s Fund (UNICEF) Joint Monitoring Programme for Water and Sanitation (JMP) provides a “sanitation ladder” to estimate access to sanitation services. This ladder classifies facilities into “unimproved”, “limited” (shared, improved facility), “basic” (private, improved facility), or “safely managed” (private, improved facility with adequate evacuation and/or treatment of excreta). Sanitation facilities are classified as “improved” when they hygienically separate excreta from human contact [13]. In addition to these technical aspects, a critical notion to consider is the *accessibility* to improved facilities. The JMP suggests the following definition of accessibility: “facilities that are close to home that can be easily reached and used when needed” [14].

In informal settlements, where shared facilities are common, access to sanitation can be undermined by security concerns, leading to unsafe defecation practices [15]. This accessibility issue disproportionately affects women due to exposure to sexual violence [16, 17], raising controversies around shared sanitation solutions and the criteria used in the JMP’s sanitation ladder. For instance, the fact that both the “safely managed” and even “basic” sanitation categories exclude, indistinctively, any amenity used by more than one family has been challenged [18]. The JMP’s definition contrasts with the more extensive definition given by the United Nations Human Settlement Programme (UN-Habitat) [3], applied when characterizing “slum” households, that is, “access to adequate sanitation in the form of a private or public toilet shared by a reasonable number of people.” Clearly, there is no one-size-fits-all solution. Experts in the field argue that different settlement “types” (defined by physical, socioeconomic, and cultural aspects) require different technical solutions [19, 20], and hence, the solutions implemented should be as varied as the settlement “types” addressed.

In the short-term, transitional and affordable sanitation solutions are urgently needed, especially in impoverished settings. These probably include on-site solutions that may or may not be shared by more than a single household. In sub-Saharan cities, on-site sanitation solutions—latrines or toilets connected to septic tanks—are already more prevalent and on the rise, being utilized by 62% of the urban population in 2020 [8]. Between 2000 and 2020, while the coverage of safely managed sanitation increased and open defecation decreased, the part of “limited” sanitation services—i.e., improved facilities that are shared by more than one household—has remained stable, covering 32% of the population [21]. This raises short-term challenges to expanding access to private (non-shared) sanitation in this region, which, by the time the SDGs were launched, required a substantial increase in investments in the WASH sector hardly achievable by low- and middle-income countries [22].

Against this background, it is crucial to investigate what specific conditions favor safe access to sanitation in situations where private facilities are not attainable in the short- or medium-term, either for financial or physical limitations. This is particularly relevant in cities like Nairobi (in Kenya), where innovative, shared sanitation solutions are rising [23]. Numerous studies focused on the health impacts of different sanitation interventions based on the JMP categories [9, 24, 25]. However, they often did not address other relevant aspects, for instance, how the location of toilets and the built environment impact access to these facilities, thus hampering their potential health benefits.

There is a lack of empirical studies assessing environmental determinants of access to shared sanitation facilities from an explicit spatial perspective, notably in informal settlements. Our study addresses this gap by investigating how the location of toilets and the configuration of the built environment relate to the perceived safety of users and the risk of diarrheal infections in two cities in West and East Africa. By nuancing our understanding of shared facilities, this research contributes to improving sanitation policies and monitoring tools, and might also inform future interventions aiming to improve access to sanitation in low-income settings. In this sense, our study aimed to determine whether the configuration of the built environment and the specific location of toilets is associated with the perceived safety to access these facilities, and whether the latter is associated with the occurrence of diarrheal infections.

## Methods

### Geographic scope

**Fig. 1:**
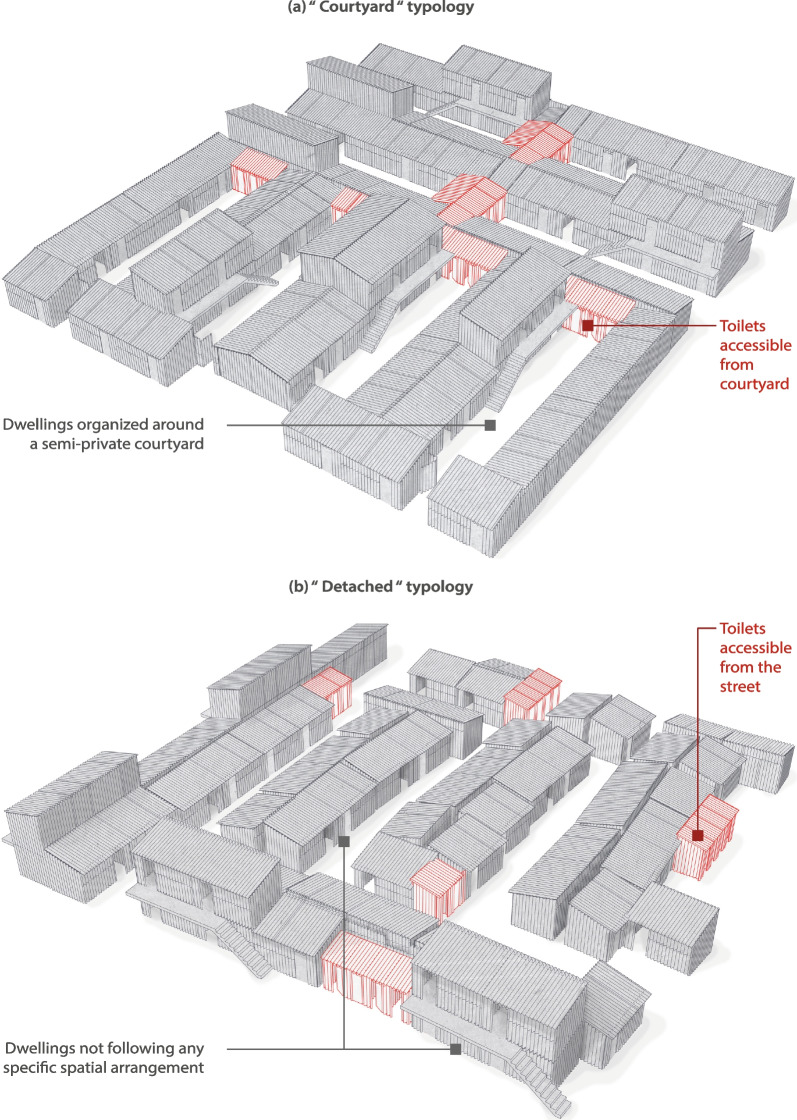
Main typologies of the spatial disposition of dwellings and toilet facilities observed in the selected informal settlements

The study was conducted in informal settlements located in Abidjan, Côte d’Ivoire and Nairobi, Kenya. We choose these two cities because they are illustrative of recent urbanization trends in sub-Saharan Africa, which has concentrated demographic growth in a limited number of locations, at the same time the lack of affordable housing in the formal sector has pushed most of the population to live in informal settlements [1, 26]. Indeed, in 2020, over half of the Ivorian and Kenyan urbanites lived in informal settlements [2], making them the predominant type of urban habitat, notably in Abidjan and Nairobi [26, 27], while posing sizeable challenges to planners in both countries regarding access to essential services such as adequate sanitation. Moreover, including study sites from distinct African regions accounts for differences in how people use sanitation facilities, as the health benefits and perception of shared facilities can vary between countries [28]. Most importantly, the two cities included in our study are characterized by a variety of urban forms [29, 30], which are the key exposure variables in this study. We sought sites with different spatial dispositions of the dwellings’ structures and of shared sanitation facilities, following two main typologies (Fig. 1): (i) a “courtyard” typology, i.e., dwellings placed around a semi-private courtyard, with one or more shared facilities located in the courtyard; and (ii) a “detached” housing typology, i.e., dwellings accessible directly from the street, with shared toilets located in the public space.

**Fig. 2:**
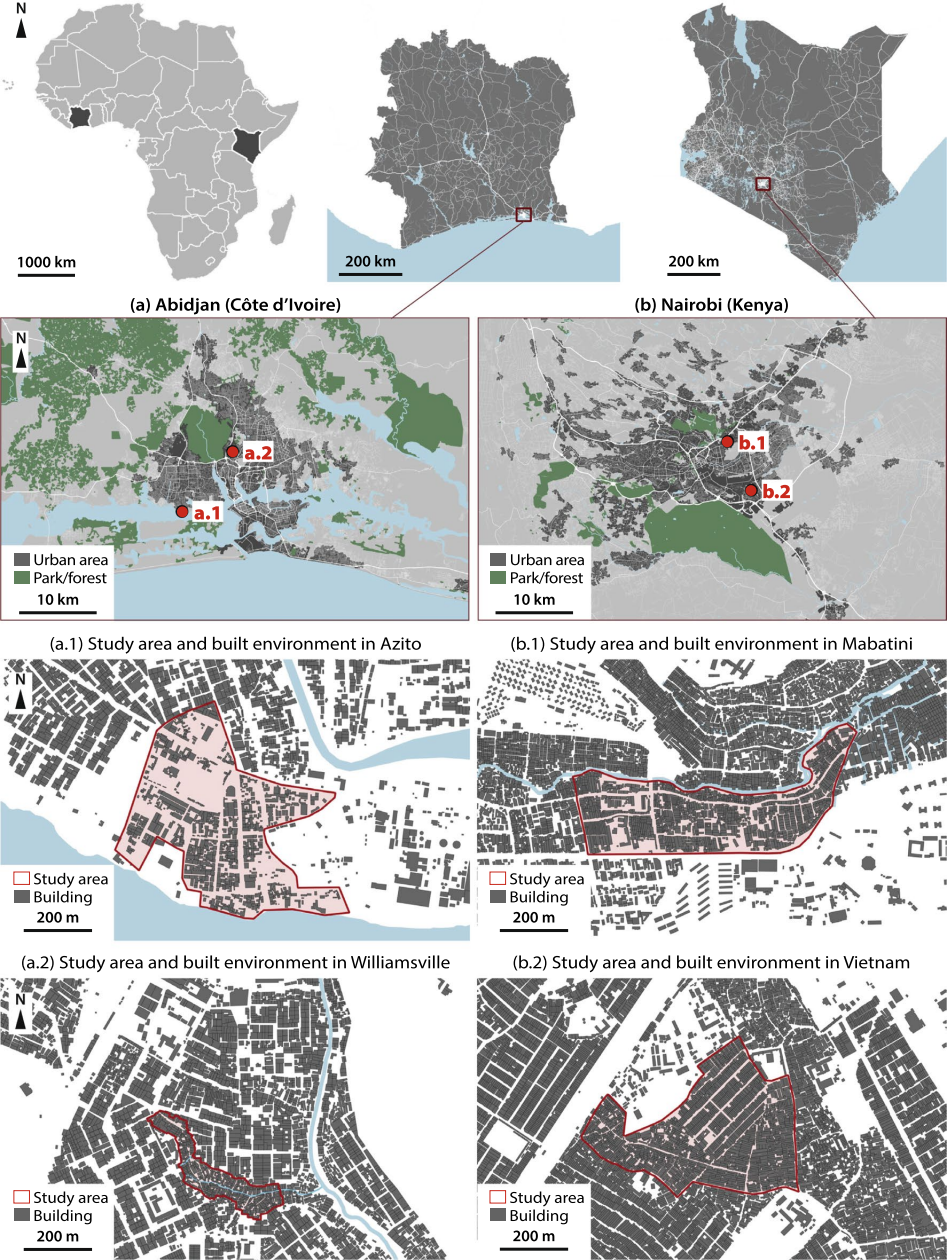
Geographic location of selected study sites (elaborated from GADM; OpenStreetMap; Ecopia Building Footprints © 2021 Ecopia Tech Corporation, Imagery © 2021 DigitalGlobe, Inc.)

Figure 2 shows the locations of the selected study sites. In Abidjan, Azito (Fig. 2, a.1), is a fishers’ village located by the lagoon, in a peripheral area of Abidjan; Williamsville (Fig. 2, a.2), is situated near to one of the main roads leading to Abidjan’s central business district (CBD). In Nairobi, Mabatini (Fig. 2, b.1), is located in a former quarry along Mathare River, 5 km away from Nairobi’s CBD; and Vietnam (Fig. 2, b.2), is located next to Nairobi’s main industrial area, in the southern periphery of the city. The four sites and their respective study perimeters were determined in collaboration with the research partners from Kenya Medical Research Institute (Nairobi) and the Centre Suisse de Recherches Scientifiques en Côte d’Ivoire (Abidjan), and community leaders (village chiefs and community health volunteers).

### Data and procedures

We used two types of data layers: sociodemographic, and settlement morphology. The former consisted of primary data collected through structured household questionnaires using the mobile application KoboCollect 1.30.1 (Kobo, Cambridge, USA). The latter consisted of maps of the buildings’ footprints of the respective sites, extracted from very high-resolution satellite imagery (± 50 cm/pixel) obtained from Ecopia [31]. To ensure accuracy, these maps were manually corrected following on-site verifications, using the geographic information software QGIS 3.10.4 (Free Software Foundation, Boston, USA).

Household data collection in Nairobi took place between July and August 2021, and in Abidjan in February 2022. In both cities, we aimed to avoid the rainy season—which, besides posing accessibility challenges, could affect the incidence of diarrhea [32] and thus bias our findings. The household questionnaires were conducted by a team of field enumerators who received a 4-day training to be familiarized with the questionnaire and the mobile applications used. Each enumerator was equipped with a tablet Galaxy Tab A 8.0 2019 (Samsung, Suwon-si, the Republic of Korea), which they used to administer the questionnaire, with the KoboCollect app. The investigators supervised all the fieldwork on-site. In total, 1147 valid surveys were obtained in Nairobi, and 567 in Abidjan; hence, meeting the quantitative requirements determined by the sample size calculation. The field enumerators were instructed to conduct the questionnaire with the head of the household, or their companion (adults only).

**Fig. 3:**
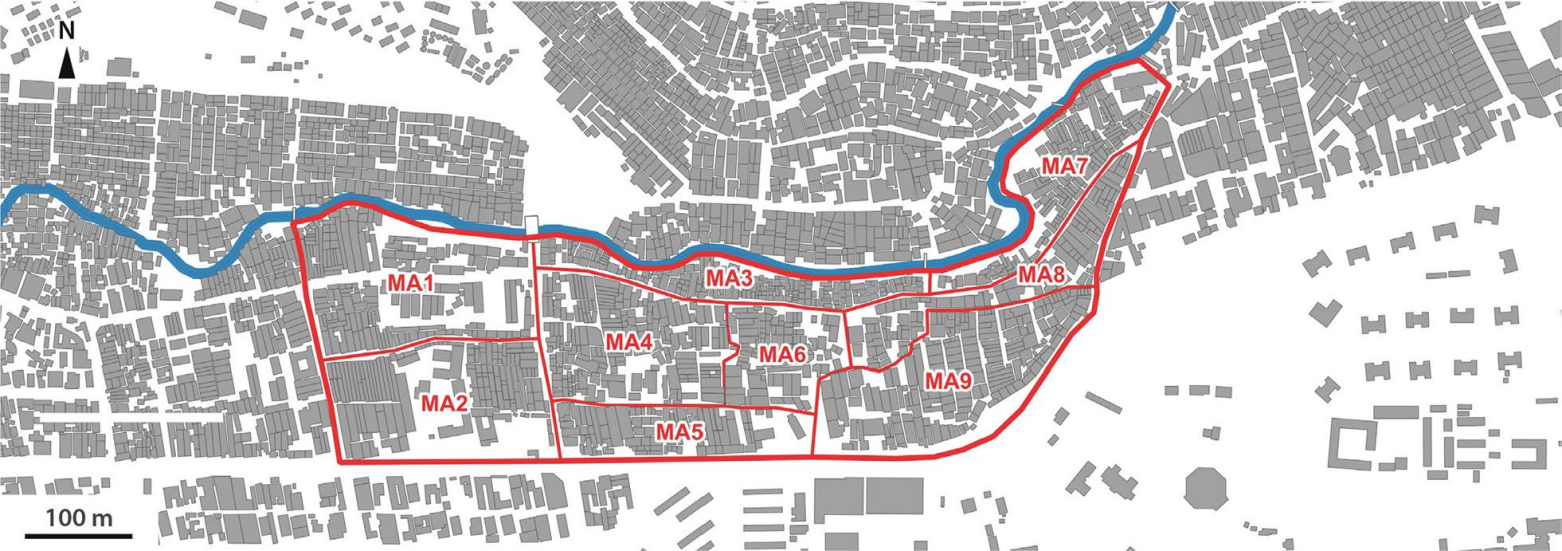
Map of Mabatini (Nairobi), with perimeters of its nine survey areas (“MA1” to “MA9”) marked in red (elaborated from Ecopia Building Footprints © 2021 Ecopia Tech Corporation, Imagery © 2021 DigitalGlobe, Inc.)

To ensure a homogenous spatial distribution of the household surveys, each study site was divided into several areas based on the number of residential buildings (see example in Fig. 3). Each area received the same number of surveys. Between two and three enumerators worked in each area, and each group was accompanied by a local resident during the fieldwork, either a community health volunteer or a community leader. The enumerators used their tablets as a navigation tool: an interactive map of each site showing all buildings’ footprints and survey areas was available on the navigation app OsmAnd 3.9 (OsmAND B.V., Amstelveen, Netherlands), which displayed their live position through the tablet’s global positioning system (GPS). To randomize the sample, the field enumerators were instructed to do a random walk, selecting one out of two addresses. Every household survey was geo-tagged, using the tablet’s GPS.

**Fig. 4:**
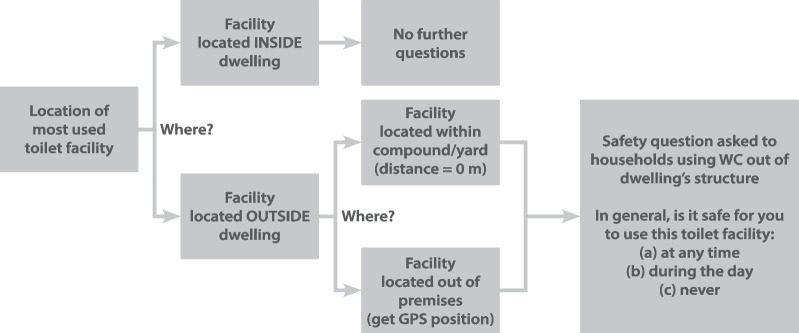
Algorithm for questions regarding toilet location and perceived safety to access the toilet

The location of toilets and the user’s perceived safety to access them were essential information to this study. Hence, the questionnaire included questions regarding these two aspects, following the algorithm shown in Fig. 4. The precise locations of toilets situated outside premises (i.e., outside the dwelling or the compound’s walls) were recorded with the tablet’s GPS, when applicable. For households having a toilet inside the dwelling, or inside the compound walls, the location attributed to the toilet was the same as the household’s location, in which case we applied a theoretical distance of 0 m.

### Health outcome of interest

Diarrhea is the principal contributor to the disease burden from WASH [12] and is an indicator commonly used to measure the health impact of water and sanitation services [33]. Building on previous cross-sectional studies in Nairobi [34] and Abidjan [35], we identified cases of diarrhea occurring in the 2 weeks preceding the survey. We used the WHO definition for diarrhea, i.e., the passage of three or more loose or liquid stools per day [36].

### Sample size

The sample size was determined by the expected prevalence of the health outcome of interest, i.e., diarrhea. Because the prevalence is usually higher amongst children under the age of 5 years than their older counterparts, we used the expected prevalence amongst children in this age group as a parameter. Moreover, diarrheal diseases represent one of the leading causes of death in under-5-year-old children [37], which justifies accounting for this specific age group. The sample size formula was given by the guidelines of Lwanga and Lemeshow for prevalence studies [38]. The parameters used were based on previous studies on diarrhea in vulnerable communities in Nairobi [34] and Abidjan [35]. The sample size (number of individuals) is given by Eq. 1:

1 $${{\varvec{n}}}_{0}\boldsymbol{ }=\boldsymbol{ }\frac{{{{\varvec{Z}}}^{2}}_{1-\propto /2}\boldsymbol{ }\times \boldsymbol{ }{\varvec{P}}\boldsymbol{ }\times \boldsymbol{ }(1-{\varvec{P}})\boldsymbol{ }\times \boldsymbol{ }{{\varvec{D}}}_{{\varvec{e}}{\varvec{f}}{\varvec{f}}}}{{{\varvec{e}}}^{2}}$$

where ${{\varvec{n}}}_{0}$ is the total number of people theoretically required for the survey; ${{{\varvec{Z}}}^{\boldsymbol{ }}}_{1-\propto /2}$ is the critical value for the standard normal distribution corresponding to a Type I error rate of **α** (in this case, **α** = 0.05; hence ${{{\varvec{Z}}}^{\boldsymbol{ }}}_{1-\propto /2}$ = 1.96); ${\varvec{P}}$ is the expected prevalence rate in the targeted population; ${{\varvec{D}}}_{{\varvec{e}}{\varvec{f}}{\varvec{f}}}$ is the “design effect” of cluster sampling; and ${{\varvec{e}}}^{2}$ is the margin of error to be tolerated at 95% level of confidence (in this case, ${{\varvec{e}}}^{2}$ = 0.05). Given that the basic unit of the survey was the *household*, the sample size obtained from Eq. 1 was adjusted to be counted in terms of households. We rectified the unadjusted sample (${{\varvec{n}}}_{0}$) by a composite factor given by: (i) the proportion of the targeted population; (ii) average household size; and (iii) the expected valid response rate (proportion of questionnaires effectively completed, and without data entry errors). Table 1 shows the adjusted sample sizes.

**Table 1:** Estimation of minimum sample sizes in Nairobi and Abidjan

Parameter	Nairobi	Abidjan
${Z}_{1-\propto /2}$ (95% level of confidence)	1.96	1.96
Expected prevalence rate ($P$)	0.20	0.15
Design effect (${D}_{eff}$)	1.50	1.50
Margin of error ($e$^2^)	0.05	0.05
Unadjusted sample size (minimum number of individuals)	**369**	**294**
Proportion of children under the age of 5 years	0.12^1^	0.15^2^
Number of people needed given the proportion of children under 5 years	3073	1959
Average household size (number of individuals)	3.0^1^	4.5^2^
Expected valid response rate	0.9	0.9
Adjusted sample size (minimum number of households)	**1138**	**484**

^1^Parameter obtained from the 2019 Kenya Population and Housing Census

^2^Parameter obtained from the 2014 Population and Housing Census of Côte d’Ivoire

### Settlement morphology

The field of urban morphology focuses on the form of the built environment, which can be decomposed and assessed quantitatively through specific indicators [39]. These indicators can be derived from buildings’ shape, size, and orientation at different geographic scales, from the single object level to the block or city level [40]. Based on previous studies that focused on the morphology of informal settlements across the globe [41, 42], we selected a series of indicators related to the density and entropy of the built environment. Given the detailed geographic scale of our analysis, these indicators were calculated at the object (single building) and block level, as described in Table 2.

**Table 2:** List of morphological indicators, distinguished by object and block level

Variable	Level	Description/calculation
Building orientation (entropy)	Object (building)	*if Azim *_*MBR*_^*1*^ < *45°:* O_B*i*_ = Azim _MBR_ *if Azim *_*MBR*_^*1*^ ≥ *45°:* O_B*i*_ = Azim _MBR_ − 2x(Azim _MBR_ − 45)
Mean deviation with first four neighbors (entropy)	Object (building)	$\frac{|{O}_{B0}-{O}_{B1}|+|{O}_{B0}-{O}_{B2}|+|{O}_{B0}-{O}_{B3}|+|{O}_{B0}-{O}_{B4}|}{4}$
Zonal, mean deviation from neighbors (entropy)	Block (100 m radius)	Iterative calculation: for each building, get the mean deviation values of all neighbors within 100 m
Voronoi tessellation (density)	Object (building)	Voronoi tessellation cells obtained from the buildings’ footprints, generating a plot-like structure [43]
Covered area ratio (density)	Object (building)	$\frac{{Area}_{building footprint}}{{Area}_{tessellation cell}}$
Zonal, mean covered area ratio (density)	Block (100 m radius)	Iterative calculation: for each building, get the mean covered area ratio of all neighbors within 100 m
Circular compactness (density)	Object (building)	$\frac{{Area}_{building footprint}}{{Area}_{enclosing circle}}$
Zonal, mean circular compactness (density)	Block (100 m radius)	Iterative calculation: for each building, get the mean circular compactness of all neighbors within 100 m
Number of neighboring structures (density)	Block (100 m radius)	Iterative calculation: for each building, count the number of structures (building footprints) within 100 m
Altitude standardized by site (topography)	Object (building)	$\frac{{Alt}_{household}-{Alt}_{site min.}}{{Alt}_{site max.}-{Alt}_{site min.}}$

^1^Azimuth of minimum bounding rectangle (MBR): orientation of axis between 1st and 3rd quadrant of the MBR

**Fig. 5:**
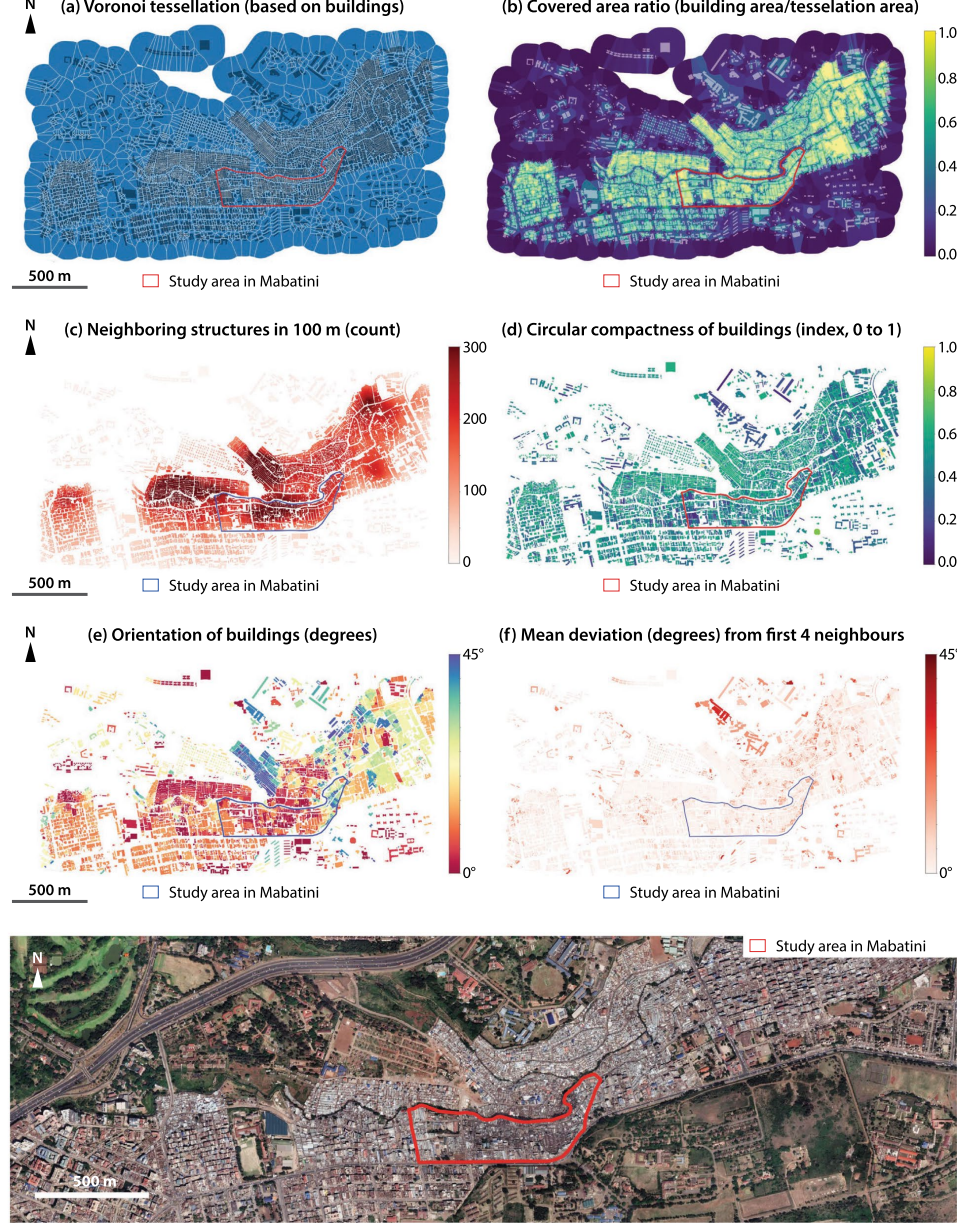
Morphological indicators used in the analysis; example of the study site in Mabatini, Nairobi (elaborated by the authors from: Google Earth; Ecopia Building Footprints © 2021 Ecopia Tech Corporation, Imagery © 2021 DigitalGlobe, Inc.)

The indicators mentioned above were calculated in Python language from the buildings’ footprints maps, using the package Momepy (version 0.5.0) [44]. We used the maps of the buildings’ footprints of the respective sites to calculate the morphological indicators—in these maps, each building footprint was represented by a single polygon. Figure 5 illustrates the morphological indicators described in Table 2 through thematic maps, showing the example of the study site in Mabatini, Nairobi. As can be seen, Mabatini is a densely occupied area—at least in terms of the number of buildings and the covered area ratio (CAR). Also, the building orientation map shows a high variation (depicted by the colors ranging from red to blue), which means the level of entropy of the built environment is relatively high. This is certainly due to the topographic complexity of the site, which used to be a quarry and has an important variation in terms of altitude between Juja road (southern perimeter of the study site) and the Mathare River (northern perimeter of the study site).

### Statistical analysis

All statistical analyses were conducted in Python language, using the packages Geopandas 0.9.0 [45], SciPy 1.7.1 [46], and Statsmodels 0.13.2 [47]. We used adjusted odds ratios (a*OR*s) to test associations between the location of the most frequently used toilet and the perceived safety to use this facility, and between perceived safety and diarrhea. Indeed, given the presence of several relevant variables for the two outcomes of interest (perceived lack of safety and diarrhea), we used two multiple logistic regression models (MLR) to obtain a*OR*s [48]—i.e., one MLR for each outcome, as shown in Table 3. All independent variables included in the MLRs had a variance inflation factor inferior to 5, thus reasonably averting issues due to multicollinearity. The a*OR*s’ significance was given by two parameters: the 95% confidence interval (*CI*) had to exclude 1, and the logistic model’s overall fit needed to be acceptable, i.e., a likelihood ratio test’s (LLR) *P* value < 0.05.

**Table 3:** Variables included in the multiple logistic regression models used to calculate adjusted odds ratios

Dependent variable	Stratification	Independent variables
[MLR model 1] Perceived lack of safety to access the most used toilet: whether the respondent representing the household considers it unsafe to access the toilet at any time	Stratum in Abidjan: *N*_1_ = 245 households (with valid answers for all 5 variables) Stratum in Nairobi: *N*_1_ = 948 households (with valid answers for all 5 variables)	•Toilet most frequently used by the household is located out of premises (exposure) •Toilet most frequently used by the household is shared with other household(s) (control) •Head of the household attained at least secondary education (control) •Respondent was female (control)
[MLR model 2] Diarrhea: whether the individual had diarrhea in the 2 weeks preceding the survey	Strata in Abidjan: *N*_2 gen pop_ = 942 individuals in the general population (living in a household with valid answers for all 7 variables)^2^ *N*_2 under 5_ = 106 individuals in the population under 5 years (living in a household with valid answers for all 7 variables)^2^ Strata in Nairobi: *N*_2 gen pop_ = 1899 individuals in the general population (living in a household with valid answers for all 7 variables)^2^ *N*_2 under 5_ = 250 individuals in the population under 5 years (living in a household with valid answers for all 7 variables)^2^	•Individual lives in a household where access to the most frequently used toilet is considered unsafe (exposure) •Individual lives in a household where the most frequently used toilet is considered ‘improved’^1^ (control) •Individual lives in a household where the most frequently used toilet is considered ‘dirty’ or ‘very dirty’ by most users (control) •Individual lives in a household with access to basic^1^ hygiene amenities (control) •Individual lives in a household where consumption of street food is frequent (control) •Head of the household where the individual lives attained at least secondary education (control)

^1^As defined by the WHO—UNICEF Joint Monitoring Programme for water, sanitation and hygiene [13]

^2^For MLR model 2, the analysis was done at individual level because the cases of diarrhea were reported at individual level. *MLR* Multiple logistic regression

Perceived safety was defined based on a structured question (Fig. 4): if the respondent declared to feel safe to use the facility at *any time* (including at night), we considered the facility in question to be perceived as “safe”. The MLR used to calculate a*OR*s for the perceived lack of safety included four variables: toilet’s location (inside or outside premises), whether it is shared with one or more household(s), respondent’s sex, and education level (potential confounders). Then, we tested associations between the perceived lack of safety and the selected morphological indicators (Table 2) through descriptive statistics and bivariate logistic regressions. We included the distance to the most frequently used toilet in both the descriptive analysis and the logistic regressions. In the former, we summarized the characteristics of the built environment around the household of: (i) those feeling *unsafe*; and (ii) those feeling *safe* to use the toilet at any time, obtaining their mean values for these two groups. To compare the two groups, the values were standardized to a common scale (0–100) and displayed in polar graphs (Fig. 7). To test the significance of the associations between each morphological indicator and safety, we ran bivariate logistic regressions, stratified by city and site within each city.

For diarrhea, the logistic regressions were stratified by city and age groups: general population in Abidjan and Nairobi, and children under the age of 5 years in Abidjan and Nairobi. In addition to the exposure of interest (lack of safety), the MLR included toilet characteristics that may affect the risk of diarrhea: whether it was considered “improved”, and its hygiene conditions. The latter was reported by a single respondent representing the household, and was based on smell, presence of personal hygiene items and/or traces of excreta. We used a 4-scale categorization (“very dirty”, “dirty”, “clean”, and “very clean”) that was recoded into a binary variable: toilets considered “very dirty” or “dirty” by 50% or more users were categorized as “dirty”. To account for reporting bias related to socio-demographic factors [49], the MLR included the education level and sex of the respondent (head of the household). To account for diarrhea risk factors that are not related to the household’s environment, we included the frequency of street food consumption (twice or more per week was considered ‘frequent’).

## Results

### Toilet location and its association with perceived safety and diarrhea

The question about safety was applicable to households using a toilet anywhere out of their dwelling, including in their compound’s yard, or a neighbor’s house. In Nairobi, 1075 households out of the 1147 interviewed used a toilet out of their dwelling, and in Abidjan, 281 households out of 567. We found a significant association between the use of toilets outside premises (outside the walls of the compound where the dwelling is located), and lack of safety (Table 4). This trend was consistently found in both cities, but the odds were much higher in Nairobi (a*OR* = 57.97) than in Abidjan (a*OR* = 3.14). Females tended to be more affected by the lack of safety than males when using a toilet facility outside premises, both in Abidjan (a*OR* = 1.62) and Nairobi (a*OR* = 1.25)—but the a*OR*s did not meet one of the significance criteria, as the respective 95% *CI*s included 1. Of relevance, sharing a toilet with one or more households was not significantly associated with the perceived safety—again, the respective 95% *CI*s included 1 and, in Nairobi, this exposure certainly did not have a sufficient variation for the statistical test to be reliable (only 15 out of 948 respondents used a private toilet).

**Table 4:** Adjusted odds ratios (a*OR*s) for perceived lack of safety to access the toilet (MLR model 1), by city

Exposure	Respondents in Abidjan (*n* = 245^1^)				Respondents in Nairobi (*n* = 948^1^)			
	a*OR*	Lower *CI* (95%)	Upper *CI* (95%)	Significance	a*OR*	Lower *CI* (95%)	Upper *CI* (95%)	Significance
Toilet shared by more than one household	1.58	0.61	4.13	Not significant	5.06e^7^	0.00	*inf*	Not significant
Household’s head with secondary education	0.54	0.27	1.09	*^2^	0.81	0.52	1.27	Not significant
Female respondent	1.62	0.88	3.16	Not significant	1.25	0.80	1.96	Not significant
**Toilet located out of premises**	**3.14**	**1.13**	**8.70**	**^2^	**57.97**	**35.93**	**93.53**	****^2^

Bold: statistically significant variables

^1^Corresponds to the number of household surveys having valid answers to all five variables included in the model

^2^The number of * indicates the significance of each *beta* coefficient resulting from the multiple logistic regression (MLR) which corresponds to the probability of the adjusted odds ratio (a*OR*) being equal to 1: *P value < 0.1, **P value < 0.05, ***P value < 0.01, ****P value < 0.001. *CI* Confidence interval

Lack of safety to use a toilet facility at any time was associated with higher odds of diarrheal infection in the general population (Table 5), even adjusting by relevant variables like the toilet’s hygiene conditions and presence of basic hygiene amenities at home (i.e., water, soap, and a hand-washing structure). This trend was consistently found in Abidjan and Nairobi (respectively, *OR* = 1.90 with 95% *CI*: 1.29–2.79, and *OR* = 1.69 with 95% *CI*: 1.22–2.34). Toilets considered “dirty” were also significantly associated with higher risks of diarrhea in both cities. In Abidjan, having basic hygiene amenities at home was significantly associated with lower risks of diarrhea.

**Table 5:** Adjusted odds ratios (a*OR*s) for cases of diarrhea in the general population (MLR model 2), by city

Exposure	Respondents in Abidjan (*n* = 942^1^)				Respondents in Nairobi (*n* = 1899^1^)			
	a*OR*	Lower *CI* (95%)	Upper *CI* (95%)	Significance	a*OR*	Lower *CI* (95%)	Upper *CI* (95%)	Significance
**Access to basic hygiene amenities**	**0.58**	**0.39**	**0.84**	***^2^	0.76	0.41	1.42	Not significant
Access to improved sanitation facility	1.27	0.88	1.85	Not significant	0.92	0.67	1.25	Not significant
Household’s head with secondary education	1.36	0.93	2.00	Not significant	1.08	0.80	1.45	Not significant
Frequent consumption of street food	1.45	0.96	2.20	*^2^	1.24	0.90	1.71	Not significant
**Toilet considered dirty**	**1.84**	**1.28**	**2.64**	****^2^	**1.57**	**1.11**	**2.22**	**^2^
**Lack of safety to use toilet**	**1.90**	**1.29**	**2.79**	***^2^	**1.69**	**1.22**	**2.34**	***^2^

Bold: statistically significant variables

^1^Corresponds to the number of individuals living in a household with valid answers to all seven variables included in the model

^2^The number of * indicates the significance of each *beta* coefficient resulting from the multiple logistic regression (MLR), which corresponds to the probability of the adjusted odds ratio (a*OR*) being equal to 1: *P value < 0.1, **P value < 0.05, ***P value < 0.01, ****P value < 0.001. *CI* Confidence interval

When analyzing the odds of diarrheal infection in children under the age of 5 years (Table 6), we found no significant association. For this age group, the only variable that showed a significant association with diarrhea was the presence of basic hygiene amenities at home, in Abidjan. Children under the age of 5 years living in a household using a toilet considered “dirty” were more likely to have diarrhea in both cities, but the *OR*s were not statistically significant. Of relevance, in Nairobi the MLR model was not reliable for this age group, as it had a likelihood ratio test resulting in a *P* value greater than 0.05.

**Table 6:** Adjusted odds ratios (a*OR*s) for diarrhea among children under the age of 5 years (MLR model 2), by city

Exposure	Respondents in Abidjan (*n* = 106^1^)				Respondents in Nairobi (*n* = 250^1^)			
	a*OR*	Lower *CI* (95%)	Upper *CI* (95%)	Significance	a*OR*	Lower *CI* (95%)	Upper* CI* (95%)	Significance
**Access to basic hygiene amenities**	**0.31**	**0.11**	**0.88**	**^2^	2.90	0.68	12.45	Model’s LLR^3^ *P* value > 0.05
Access to improved sanitation facility	2.48	0.94	6.54	*^2^	1.03	0.56	1.89	Model’s LLR^3^ *P* value > 0.05
Household’s head with secondary education	0.98	0.34	2.78	Not significant	0.87	0.49	1.52	Model’s LLR^3^ *P* value > 0.05
Frequent consumption of street food	1.49	0.51	4.32	Not significant	1.72	0.93	3.18	Model’s LLR^3^ *P* value > 0.05
Toilet considered dirty	2.49	0.93	6.67	*^2^	1.72	0.87	3.38	Model’s LLR^3^ *P* value > 0.05
Lack of safety to use toilet	0.77	0.24	2.46	Not significant	0.88	0.44	1.75	Model’s LLR^3^ *P* value > 0.05

Bold: statistically significant variables

^1^Corresponds to the number of individuals living in a household with valid answers to all seven variables included in the model

^2^The number of * indicates the significance of each *beta* coefficient resulting from the multiple logistic regression (MLR), which corresponds to the probability of the adjusted odds ratio (a*OR*) being equal to 1: *P value < 0.1, **P value < 0.05, ***P value < 0.01, ****P value < 0.001. *CI* Confidence interval

^1^*LLR* likelihood ratio test

### Morphological characteristics of safe and unsafe settings

**Fig. 6:**
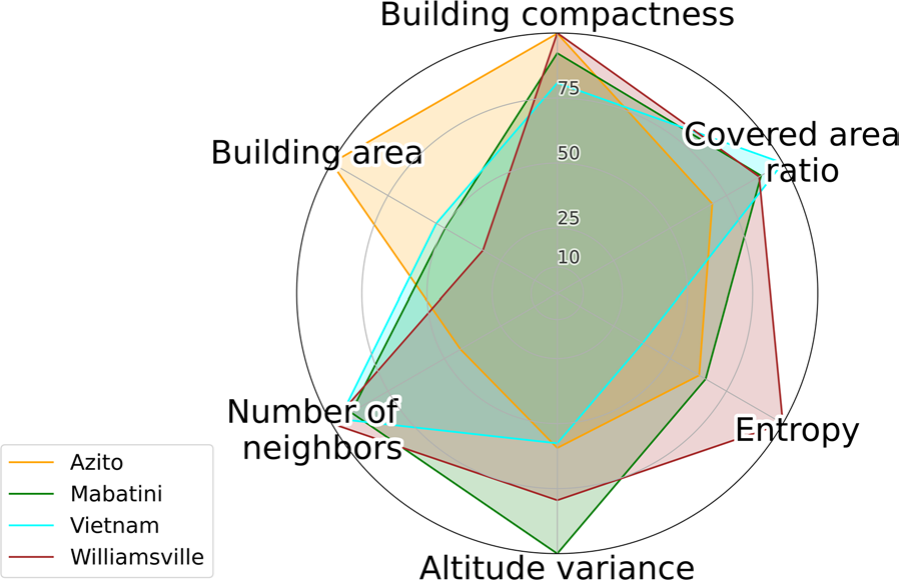
Standardized morphological indicators

**Fig. 7:**
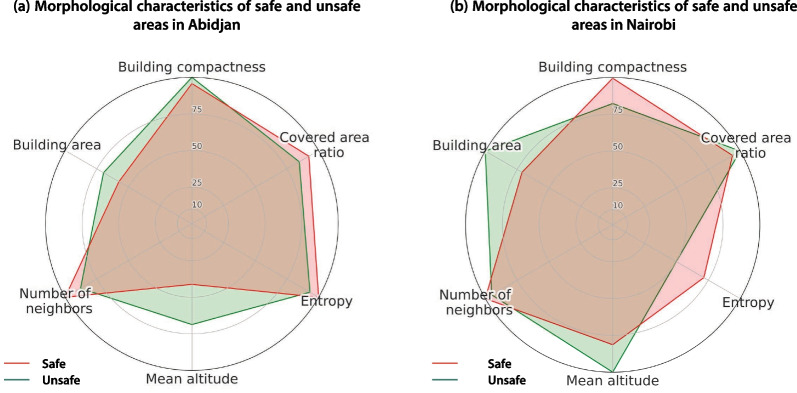
Aggregated, morphological indicators by group (“safe” and “unsafe” situations) and city

We found different levels of perceived safety to use toilets in the four study sites (Table 7), which, in turn, had different settlement morphologies. The polar graphs in Figs. 6 and 7 show descriptive statistics for the morphological indicators in different contexts. Figure 6 shows the standardized indicators calculated from all buildings in each site, giving an overview of their general morphology. Figure 7 shows the standardized indicators of the buildings where the participants lived, disaggregated by city and distinguished by “safe” and “unsafe” situations. Globally, households located in larger buildings and in higher locations in terms of topographic elevation were associated with safe access to toilets. On the other hand, households located in areas more congested—with numerous neighboring structures—and more entropic—with higher deviations in orientation between neighbors—were associated with lack of safety to access toilets. Some indicators differed between cities; namely, building compactness was associated with lack of safety in Nairobi, but not in Abidjan, while the covered-area ratio was associated with lack of safety in Abidjan, but not in Nairobi.

**Table 7:** Perceived safety in the study sites

Site	Respondents feeling unsafe (%)
Azito (Abidjan)	12.5
Williamsville (Abidjan)	28.6
Mabatini (Nairobi)	46.7
Vietnam (Nairobi)	2.7

Two sites from distinct cities, namely Mabatini (Nairobi) and Williamsville (Abidjan), presented similar morphological features (Fig. 6), such as a complex topography, with important variations in elevation, and a relatively entropic built environment, with numerous small buildings close to one another. Also, the two sites had a relatively high proportion of respondents feeling unsafe to use a toilet (Table 7): 47% in Mabatini (Nairobi) and 29% in Williamsville (Abidjan), against 3% in Vietnam (Nairobi) and 13% in Azito (Abidjan).

**Fig. 8:**
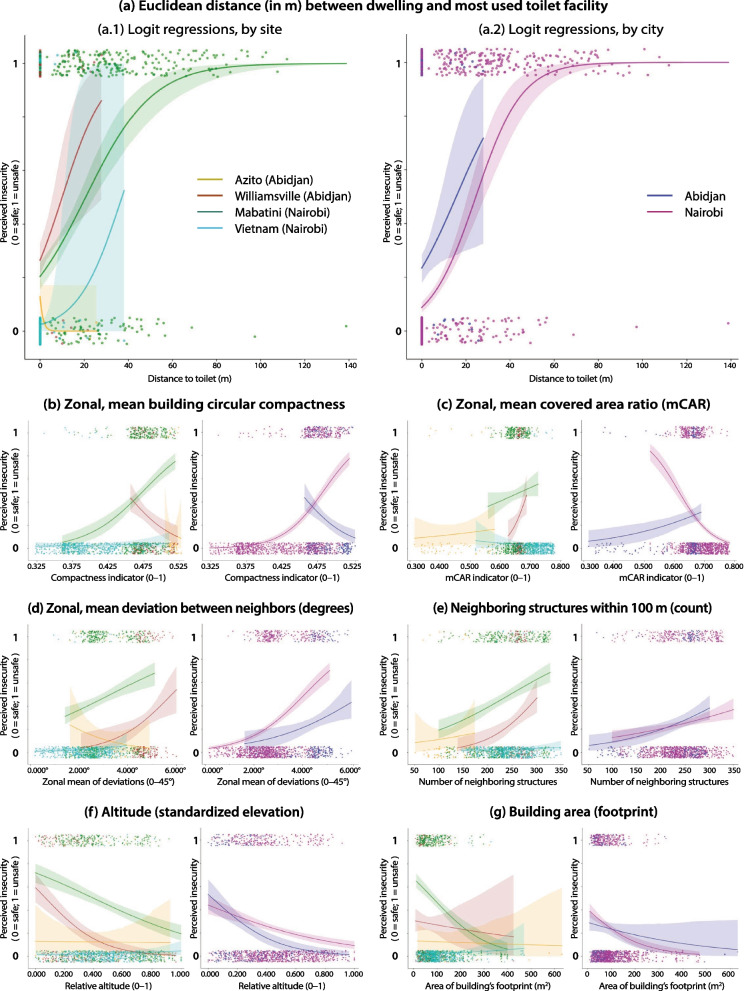
Scatterplots of bivariate logistic regressions between perceived safety to access toilets and selected morphological indicators

Figure 8 shows the scatterplots of the bivariate regressions, stratified by site (on the left) and by city (on the right), and Table 8 shows the individual model parameters for each regression. Each observation corresponds to a single surveyed household, and the morphological indicators correspond to those of the building where the household is located. Three morphological indicators—circular compactness, covered area ratio, and deviation—consisted of a zonal statistic, i.e., for each building, we calculated the mean values of all neighbors within 100 m.

**Table 8:** Model parameters of the bivariate logistic regressions, by variable and city

Variable	City	*P* value	LLR* P* value	Pseudo *R*^2^
Building area (footprint)	Abidjan	0.201	0.153	0.01
	Nairobi	< 0.001	< 0.001	0.04
Number of neighboring structures within 100 m	Abidjan	0.001	< 0.001	0.04
	Nairobi	< 0.001	< 0.001	0.01
Zonal, mean building circular compactness	Abidjan	< 0.001	< 0.001	0.06
	Nairobi	< 0.001	< 0.001	0.26
Zonal, mean covered area ratio	Abidjan	0.008	0.002	0.03
	Nairobi	< 0.001	< 0.001	0.11
Zonal, mean deviation from neighbors	Abidjan	0.006	0.003	0.03
	Nairobi	< 0.001	< 0.001	0.09
Altitude (standardized elevation)	Abidjan	< 0.001	< 0.001	0.09
	Nairobi	< 0.001	< 0.001	0.03
Euclidean distance to toilet facility	Abidjan	0.009	0.008	0.02
	Nairobi	< 0.001	< 0.001	0.37

Overall, the logistic regressions corroborate the findings shown in the polar graphs for both cities. The most significant result can be seen graphically in Fig. 8a: the distance to the toilet facility was patently associated with safety in Nairobi (*P* < 0.001, pseudo *R*^2^ = 0.37). Indeed, beyond ± 30 m between the household and the facility, people were very likely to feel unsafe to use the facility. The same effect was observed in Abidjan, which was also statistically significant (*P* = 0.009), but much less important in terms of explaining variance in the outcome (pseudo *R*^2^ = 0.02). Another variable that had an important association with safety was the circular compactness of the buildings (Fig. 8b). In Nairobi, it was significantly associated with the lack of safety (*P* < 0.001, pseudo* R*^2^ = 0.26), while in Abidjan, on the contrary, it was associated with a feeling safety (*P* < 0.001, pseudo *R*^2^ = 0.06). All the other variables, except for building area in Abidjan, also had a statistically significant association with safety (*P* < 0.05) but did not attain a pseudo-*R*^2^ value beyond 0.11—and thus explained very little of the variance in the outcome of interest. In general, the morphological indicators performed better in Nairobi than in Abidjan.

## Discussion

### Towards criteria to define safe settings for shared sanitation

Shared, on-site sanitation is among the most used types of infrastructure in sub-Saharan cities [8]. However, there are concerns regarding their actual health benefits, notably the prevention of diarrheal infections, due to issues related to the management of excreta [18] and exposure to violence [15]. The WHO Guidelines on Sanitation and Health [10] acknowledge that shared sanitation solutions should be considered when private facilities are not feasible, either for financial limitations or for lack of physical space, notably in crowded informal settlements. In such situations, though, WHO emphasizes the importance of identifying a safe location and access route to the facility, if applicable. In fact, in line with previous investigations [15–17], our results suggest that lack of safety is a major obstacle to accessing shared sanitation, especially among women.

Although normative guidelines on sanitation facilities such as those put forward by WHO provide detailed technical recommendations, they often lack clear recommendations regarding the criteria to define a “safe location” for shared facilities, notably in the context of informal settlements. Current guidelines generally focus on toilet design and technical aspects related to the safe contention and treatment of excreta [10, 50, 51], and only marginally address issues related to the perceived safety when accessing toilets. There are some exceptions [52], but there is still a lack of evidence based on empirical, spatially explicit analyses. This study addressed this knowledge gap by putting forward specific risk factors related to the toilet location—whether inside or outside the compound’s walls—and the spatial configurations of the built environment (i.e., morphological aspects).

### The physical morphology of safe settings: empirical observations

Several aspects related to the built environment’s configuration were associated with the perceived safety to access sanitation facilities. The most significant was the toilet’s location—whether inside or outside a compound’s walls. Indeed, privacy plays a key role in the quality and safety of sanitation facilities [53], thus locating such facilities in a private or semi-private location—e.g., compound’s yard, inside walls—is an efficient way to ensure safety and, naturally, users’ convenience. On the contrary, people relying on toilets situated in public locations were more likely to feel unsafe to use them in both cities, even adjusting by potential confounders such as the respondent’s sex (Table 4). Moreover, our results suggest that location is a more significant determinant of safety than sharing a toilet with other households, per se—which raises questions regarding the JMP’s current definition of “basic” sanitation. The risk of feeling unsafe significantly increased with the distance travelled: beyond approximately 30 m, it was very likely that the person felt unsafe to access the toilet. According to informal discussions with residents of the study sites, the main reason for such perceived lack of security was the fear of being harassed or mugged on their way to the facility, or even inside the facility, especially at night. This corroborates findings of previous studies in similar contexts, where social vulnerability and violence significantly affect access to sanitation, notably among women [15]. In Nairobi, most crimes committed in informal settlements happen in the evening or at early night, consisting mostly of robbery and mugging, but also sexual violence [54, 55], which could explain the fear to go to a toilet at night in our study sites. In Abidjan, chronic poverty and social exclusion has led to the rise of brutal assaults by youngsters (known as “microbes”), which has raised a generalized concern in the population [56].

Regarding the spatial disposition and form of buildings, several indicators were associated with the lack of safety in Nairobi and in Abidjan: the mean deviation with neighboring structures within a 100 m radius, the number of neighbors within a 100 m radius, and the relative altitude in each site. The fact that common trends were observed in sites of distinct African regions corroborates the idea that settlement morphology is a key determinant of the perceived safety to access shared facilities. Indeed, the two sites with the highest rate of perceived lack of safety, namely, Mabatini and Williamsville, had common characteristics: amongst the four selected sites, these two had the most entropic built environment, with a high concentration of buildings within a 100 m radius, and smaller footprints (Fig. 6). Moreover, these two sites were in areas with high variations in elevation, and the relative altitude played a significant role in increasing safety: respondents living in higher locations felt safer than those living in lower locations. The higher locations were closer to the main streets and well-illuminated areas, which certainly played a role—night-time illumination is critical to enhance safety [52].

In the logistic regressions, the morphological indicators generally performed better in Nairobi than in Abidjan. This may be explained by statistical effects given by the considerably lower number of outcomes (respondents feeling unsafe) in Abidjan. The fact that housing units in Abidjan are often organized in a “courtyard” typology [29], with shared toilets often placed in semi-private areas or even inside the dwelling, certainly affected the perceived safety. In this sense, the spatial typology of informal settlements in Abidjan may foster safer access to sanitation. Otherwise, almost all indicators were statistically significant in both sites, with *P* values below 0.05, but explained very little of the variation in safety, with pseudo-*R*^2^ seldom higher than 0.1. This is not unusual in such study designs, given the “noise” of environmental variables and the complexity of the studied ecosystems [57].

The physical morphology of informal settlements could be an indirect predictor—not necessarily a causal factor—of diarrhea and, more broadly, general health and well-being. As other authors argued, there are specific features of the built environment that can be proxy indicators of social and economic vulnerability [41, 58, 59]. With our study, we emphasize the need for further research on the physical form of impoverished settlements and its potential implications for public health.

### Perceived safety to access sanitation facilities and risk of diarrheal infections

The a*OR*s showed a consistent association between the lack of safety to access toilets and the risk of diarrheal diseases in the general population, both in Nairobi and in Abidjan (Table 5). We hypothesize that settlement morphology indirectly affects the risk of diarrhea, by facilitating—or hindering—access to sanitation facilities. Should people feel unsafe to access proper toilets, notably women, they may recur to alternatives such as buckets of plastic bags, which are much less safe in terms of the containment of excreta [15], hence entailing the threat of diarrheal infections. Indeed, in such situations there is an increased exposure to potential fecal–oral infection pathways [60], which might explain the higher risk of diarrhea in the general population, amongst those feeling unsafe to access a toilet. Among children under the age of 5 years, however, this association between lack of safety and diarrhea was not observed. We may question whether such a relation is even relevant for this age group, given that young children are not necessarily users of the toilets analyzed, and certainly recur more often to home-based solutions (diapers or bucket). If not handled properly, the latter constitute potential sources of contamination, regardless of the safety and quality of toilets commonly used by the household members. Toilet hygiene, however, was consistently associated with higher risks of diarrhea across cities and age groups (although not always significant), which might be explained by pathogens spread from “dirty” toilets to the household.

Our results suggest that, ultimately, the health benefits of sanitation infrastructures in informal settlements rely on aspects beyond the facilities themselves. Besides the toilets’ availability and hygiene conditions, their specific location seems just as important to prevent diarrheal infections. In our case studies, the spatial context was more significantly associated with diarrhea than whether the facility type was “improved”, thus reminding an old debate on the reliability of such categories purely based on technical (design) aspects of the toilet [61]. There are aspects related to the specific settlement’s morphology that are associated with the perceived safety in accessing those facilities, and this may have significant health implications. These findings resonate with a recent ecological study conducted in Côte d’Ivoire, which showed that specific landscape features related to dense, deprived settlements were a more accurate predictor of diarrhea in Ivorian urban areas than the sole availability of sanitation services [62]. In fact, although the availability of sanitation services represents a theoretical improvement, in practice, the health benefits of these improved services may be null if the targeted populations do not feel safe to use them.

In the recent past, there have been debates on the actual health impacts of WASH interventions, which are not always clearly detected [11, 25]. This led to discussions and improvements on the ways to measure access to WASH and exposure to environmental contamination, looking at coverage at communal level, on top of household-level observations [63]. Of note, these discussions on the potential health benefits of WASH could be enriched by adding an explicit spatial dimension. Indeed, further research is needed to better understand how the health impacts of sanitation interventions relate with local spatial conditions, in different social-ecological contexts. Such investigations can be useful to inform future interventions and policies.

### Policy implications: revisiting normative definitions and monitoring indicators

There is no consensus on the exact definition of “basic” sanitation, and there are growing concerns on the current pace of advancement toward SDG 6 [8]. For instance, attaining SDG 6 can be particularly challenging in settings marked by rapid urban and demographic growth, with a high prevalence of poverty. In the short-term, such contexts require a variety of solutions that are suitable and affordable to the different social and spatial contexts. In these cases, innovation and adaptation of sanitation solutions are not an option, and there are interesting examples, notably in Nairobi, of how on-site sanitation systems could work. However, these adaptations often clash with normative definitions, notably those of the JMP. From an urban health perspective, there is still little evidence regarding the trade-off between affordable, short-term solutions that would be classified as “limited” sanitation (for they are shared), and more desirable, but also more expensive, long-term solutions that would be classified as “basic” or “safely managed”. With 49% of the population in sub-Saharan Africa still relying on unimproved facilities or open defecation [8], this question is certainly relevant if we want to expand access to improved sanitation at a faster pace.

The discussion on the definition of “basic” infrastructures is essential, as it determines the key indicators used to monitor advancements in social development and infrastructural projects, notably the SDGs. In fact, the targets and indicators of SDG 6 are based on the JMP service ladder, which does not account for the location of sanitation facilities. However, the JMP acknowledges that “using a facility located on premises may be more important for health and well-being than whether the facility is shared with other households”, and recommends including a question about the location of sanitation facilities [64]. Given the evidence put forward by this study, policymakers should consider toilet location as a parameter to define “basic” sanitation, and include this information in monitoring frameworks. Important survey programs, such as the Demographic and Health Surveys (DHS) have already included a specific question about the toilet’s location. However, this is not the case for some national censuses, notably in Kenya. Indeed, the 2019 Population and Housing Census [65] did not include any question regarding toilet location.

### Study limitations

The perception of safety is subjective, varying between individuals, and these differences can be further accentuated when analyzing distinct regions. To account for eventual regional differences, we stratified the statistical analyses by city. Regarding the individual dimension of perceived safety, the goal was not to estimate a precise measure of lack of safety in each site and city, but rather to identify global trends and associations with the built environment—which was possible thanks to the sufficiently large sample sizes in both cities.

Regarding the risk of diarrhea, like in other studies based on self-reporting, the quantification of cases in this study was prone to reporting bias [66]. We used a recall period of 2 weeks, in line with the DHS [67] and the Nairobi Cross-Sectional Slums Surveys [34]. Although a 2-week recall period is relatively long when compared to other cross-sectional studies on diarrhea, reducing this period would have substantially decreased the study’s power, considering that the occurrence of diarrhea was measured only once.

Finally, the specific etiology of the observed diarrheal infections by bacteria or viruses was not addressed, as this was not the primary goal of the study. We acknowledge that, among the cases detected, some infections might have occurred outside the community’s boundaries; hence, were not related to the built environment in the study area. Moreover, the cross-sectional study design employed here cannot establish any causal relations—while highly relevant for identifying potential risk factors, including the perceived safety to access sanitation facilities and their hygiene conditions. Although this relation may be intuitive, and previous studies have reported that the lack of safety leads to hazardous defecation practices in other contexts, we could not demonstrate any causal relation in our study.

## Conclusions

Until “safely managed” sanitation—as defined by the JMP—is universally accessible (and affordable), shared sanitation solutions should be considered in some circumstances, notably, where the available space is scarce and financial resources are limited. In such situations, it is imperative to understand in what conditions shared sanitation can be safe. Our findings suggest that there are environmental determinants of the perceived safety to access toilets in the context of informal settlements. Toilets located outside the premises were often perceived as less safe than those located within the premises, while certain aspects of the built environment exacerbated the perceived lack of safety, notably the entropy of buildings, the density of structures, and the relative elevation.

Taken together, the built environment’s configuration and the specific location of sanitation facilities may indirectly affect their health benefits, by facilitating or hampering access to these facilities. In fact, our findings showed a significant association between the perceived lack of safety to access toilets and the odds of diarrhea in the general population. Hence, it is crucial to ensure the privacy and security of sanitation facilities by placing them in adequate locations. Existing normative definitions “basic” and “safely managed” sanitation rightly emphasize the importance of the facility’s design (“improved” or “unimproved”) and maintenance (proper management of excreta and overall hygiene conditions) to prevent threatening diarrheal infections. However, such normative definitions should address more explicitly the facility’s location (which may be more relevant to determine safety than sharing a facility per se). Similarly, existing sanitation guidelines should include explicit recommendations regarding the choice of location of sanitation facilities to ensure safety of access.

## Data Availability

Geo-referenced household data: the “raw”, geo-referenced household datasets analyzed during the current study are not publicly available due to concerns of privacy and protection of anonymity but are available from the corresponding author on reasonable request, given that ethical concerns are properly addressed. An anonymized version of the data allowing to replicate the analysis, can be found here: https://github.com/ceat-epfl/sanitation-informal-settlements. Building footprint data: the data that support the findings of this study are available from Ecopia but restrictions apply to the availability of these data, which were used under license for the current study, and so are not publicly available. Data are, however, available from the authors upon reasonable request and with permission of Ecopia.

## Abbreviations

CAR: Covered Area Ratio
CBD: Central business district
*CI*: Confidence interval
DHS: Demographic and Health Surveys
GPS: Global Positioning System
JMP: World Health Organization/United Nations Children’s Fund Joint Monitoring Programme for Water and Sanitation
LLR: Likelihood ratio test
MLR: Multiple logistic regression
a*OR*: Adjusted odds ratio
SDG: Sustainable Development Goal
UN-Habitat: United Nations’ Human Settlement Programme
UNICEF: United Nation’s Children Fund
WASH: Water, sanitation and hygiene
WHO: World Health Organization
